# Mortality Prediction after the First Year of Kidney Transplantation: An Observational Study on Two European Cohorts

**DOI:** 10.1371/journal.pone.0155278

**Published:** 2016-05-06

**Authors:** Marine Lorent, Magali Giral, Manuel Pascual, Michael T. Koller, Jürg Steiger, Katy Trébern-Launay, Christophe Legendre, Henri Kreis, Georges Mourad, Valérie Garrigue, Lionel Rostaing, Nassim Kamar, Michèle Kessler, Marc Ladrière, Emmanuel Morelon, Fanny Buron, Dela Golshayan, Yohann Foucher

**Affiliations:** 1 EA 4275 SPHERE—Biostatistics, Clinical Research and Pharmaco-Epidemiology. Nantes University, Nantes, France; 2 Transplantation, Urology and Nephrology Institute (ITUN)—INSERM U1064, CHU Nantes, Nantes, France; 3 CIC Biotherapy, CHU Nantes, Nantes, France; 4 Transplantation Center, CHUV and University of Lausanne, Lausanne, Switzerland; 5 Basel Institute for Clinical Epidemiology and Biostatistics, University Hospital Basel, Basel, Switzerland; 6 Transplantation Immunology and Nephrology, University Hospital Basel, Basel, Switzerland; 7 Service de Transplantation Rénale et de Soins Intensifs, Hôpital Necker, APHP Paris, Paris, France; 8 Universités Paris Descartes et Sorbonne Paris Cité, Paris, France; 9 Service de Néphrologie-Transplantation, Hôpital Lapeyronie, Montpellier, France; 10 Service de Néphrologie, HTA, Dialyse et Transplantation d'Organes, CHU Rangueil, Toulouse, France; 11 Université Paul Sabatier, Toulouse, France; 12 Service de Transplantation Rénale, CHU Brabois, Nancy, France; 13 Service de Néphrologie, Transplantation et Immunologie Clinique, Hôpital Edouard Herriot, Lyon, France; Centre for Inflammation Research, UNITED KINGDOM

## Abstract

After the first year post transplantation, prognostic mortality scores in kidney transplant recipients can be useful for personalizing medical management. We developed a new prognostic score based on 5 parameters and computable at 1-year post transplantation. The outcome was the time between the first anniversary of the transplantation and the patient’s death with a functioning graft. Afterwards, we appraised the prognostic capacities of this score by estimating time-dependent Receiver Operating Characteristic (ROC) curves from two prospective and multicentric European cohorts: the DIVAT (Données Informatisées et VAlidées en Transplantation) cohort composed of patients transplanted between 2000 and 2012 in 6 French centers; and the STCS (Swiss Transplant Cohort Study) cohort composed of patients transplanted between 2008 and 2012 in 6 Swiss centers. We also compared the results with those of two existing scoring systems: one from Spain (Hernandez et al.) and one from the United States (the Recipient Risk Score, RRS, Baskin-Bey et al.). From the DIVAT validation cohort and for a prognostic time at 10 years, the new prognostic score (AUC = 0.78, 95%CI = [0.69, 0.85]) seemed to present significantly higher prognostic capacities than the scoring system proposed by Hernandez et al. (p = 0.04) and tended to perform better than the initial RRS (p = 0.10). By using the Swiss cohort, the RRS and the the new prognostic score had comparable prognostic capacities at 4 years (AUC = 0.77 and 0.76 respectively, p = 0.31). In addition to the current available scores related to the risk to return in dialysis, we recommend to further study the use of the score we propose or the RRS for a more efficient personalized follow-up of kidney transplant recipients.

## Introduction

Kidney transplantation (KT) is known to be the treatment of choice for end-stage renal disease. Population analyses have demonstrated that KT recipients (KTR) have a lower mortality than patients on dialysis awaiting transplantation [1–4]. However, on an individual level, the mortality risk varies between patients, resulting in a heterogeneity of the benefit in relation to transplantation [5]. This is particularly important with regard to the ageing of recipients, as in the United States for instance where the proportion of candidates on the KT waiting list over the age of 65 years has increased during the past decade from 10 to 18% [6].

The stratification of recipients according to their mortality risk could be helpful to clinicians for personalizing medical management by adapting outpatient follow-up frequency. As an example, we currently proceed to such adaptation by video-conferencing in the frame of a French multicenter randomized study [7], in which the visits frequency is driven by the long-term risk of return to dialysis evaluated by a decision making tool so-called: “Kidney Transplant Failure Score (KTFS)” and computed at 1-year [8]. We voluntarily built the KTFS at one year post transplantation since it seems difficult to propose such adaptation within the first months after transplantation when numerous clinical events can frequently occur (infections, acute rejection episodes, treatment adaptations, etc.). In addition to the prediction of the risk of return to dialysis, we hypothesized that the combined evaluation with the risk of long-term mortality could improve the risk stratification for a better medical follow-up adaptation.

In 2009, Hernandez et al. proposed such a risk score computable at 1-year post transplantation for mortality prediction with a C-index value at 0.74 (95%CI = [0.70, 0.77]) for a prognostic at 3 years since the first anniversary of the transplantation [9]. This retrospective study was conducted on Spanish patients receiving a KT in 1990, 1994, 1998 and 2002. This score took into account 8 variables: recipient age at the transplantation, history of diabetes and hepatitis C virus (HCV), new onset diabetes after transplantation (NODAT), 1-year serum creatinine, 1-year 24h-proteinuria and maintenance immunosuppressive therapy with Tacrolimus or Mycophenolate Mofetil (MMF) within the first year of transplantation. Nevertheless, to our knowledge, there is no publication concerning an external validation of this score upon other cohorts.

In the United States, Baskin-Bey et al. [10] have developed the Recipient Risk Score (RRS) based on 4 recipient characteristics: recipient age, history of diabetes, cardiac angina and duration on dialysis therapy. Compared to other pre-transplant scores [11–15], it currently presents the highest capacities for mortality prediction with a C-statistic at 0.78 for a prognostic at 5 years since the transplantation [16]. Nevertheless, because the RRS only considers recipient characteristics at the time of transplantation, one can expect that the addition of donor and transplantation characteristics within the first year post transplantation could improve its capacities to predict the long term mortality.

The primary objective of our study was to develop an alternative mortality scoring system calculated at 1-year post transplantation. The secondary aim was to study its prognostic capacities from two Western European cohorts and to compare the corresponding prognostic capacities with the ones of the both scoring systems proposed by Hernandez et al. [9] and Baskin-Bey et al. [10].

## Materials and Methods

### Study population consisting of two distinct cohorts

To conduct this study, we used the prospective French multicentric DIVAT cohort (Données Informatisées et VAlidées en Transplantation, www.divat.fr). About one third of the kidney transplantations performed today in France are included in the DIVAT cohort. Codes were used to assure patients anonymity and blind assay. The “Comité National Informatique et Liberté” approved the study (N° CNIL 891735) and written informed consent was obtained from the participants. Patients transplanted between 2000 and 2012 in 6 French University Hospitals (Nantes, Necker, Nancy, Toulouse, Montpellier and Lyon) and who remained alive with a functioning graft after one year post transplantation were included in the study. Only adults receiving a single KT and maintained under Calcineurin inhibitors and MMF for maintenance therapy after transplantation were considered. Patients with multi-organ transplantation or with missing values for at least one variable of the scores proposed by Hernandez et al. and Baskin-Bey et al. were excluded. 3439 KTR were included. Patients non-included in the study due to missing values were compared to the ones included (S1 Table): the two groups appeared to be comparable.

We also used the prospective multicenter Swiss Transplant Cohort Study (STCS, www.stcs.ch) [17]. Patients transplanted between 2008 and 2012 in all 6 Swiss University Hospitals (Basel, Bern, Geneva, Lausanne, Saint-Gall and Zurich) were included. The other inclusion criteria were the same than those used for the French DIVAT cohort. 800 KTR were included. Since 2008, the STCS cohort has included the totality of the kidney transplantations carried out in Switzerland.

**Fig 1:**
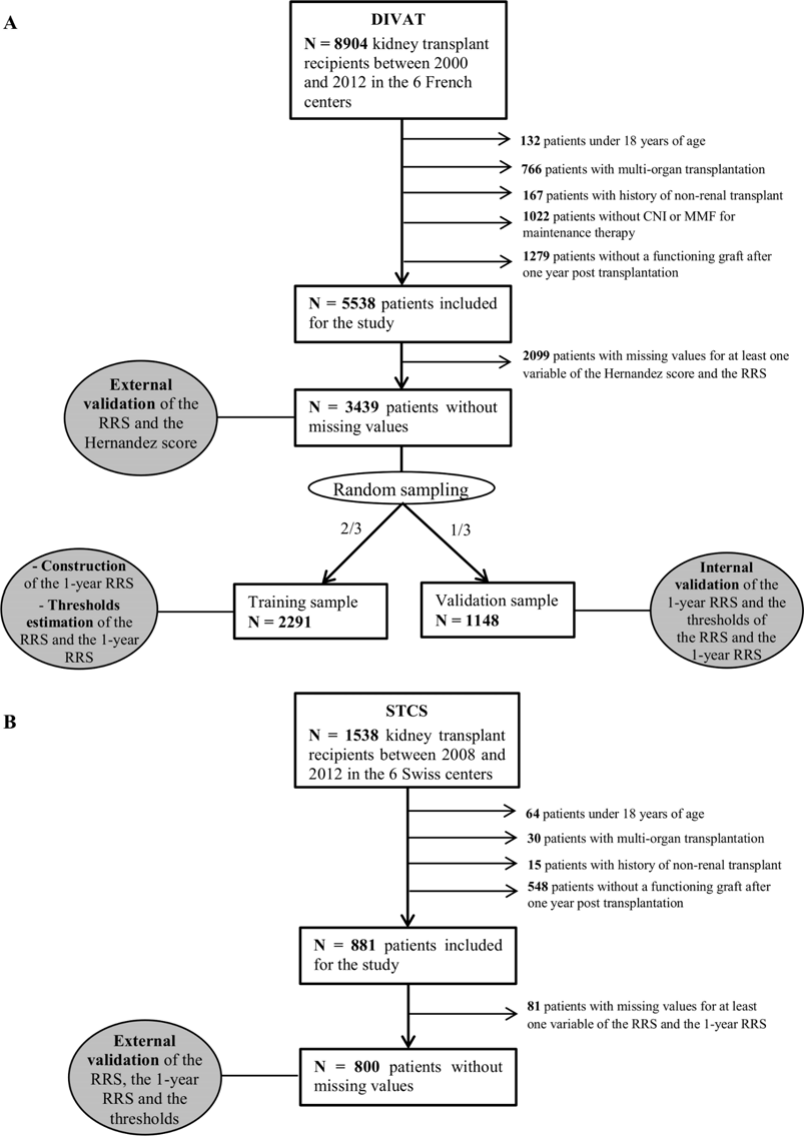
Flowcharts presenting the steps of the patient inclusion in (A) the French DIVAT cohort and (B) the Swiss STCS cohort.

The flowcharts of each cohort are presented in Fig 1.

### Available data

For the French DIVAT cohort, recipient variables were collected at the time of transplantation: gender, age, body mass index (BMI), time between the registration on the waiting list and transplantation, time duration on dialysis before transplantation, previous transplants, primary kidney disease, dialysis modality, cytomegalovirus (CMV) serology, HCV serology, past history of either diabetes, high blood pressure, cardiovascular event, angina, dyslipidaemia and neoplasia. Donor variables extracted were: gender, age, donor type, cause of death, serum creatinine, and serology (CMV, EBV and HCV). Available transplant data were: cold ischemia time and number of Human Leukocyte Antigen (HLA) incompatibilities (A-B-DR). Furthermore, the following variables were collected within the first year post transplantation: NODAT, maintenance therapy with Tacrolimus, 1-year serum creatinine and 1-year 24h-proteinuria.

For the Swiss STCS cohort, only variables taken into account in the RRS and the alternative 1-year prognostic score were extracted from the database.

Note that donor and recipient ethnicities were not available in the two databases and could therefore not be taken into account.

### Data analysis

#### The construction of a scoring system at 1-year post transplantation

The DIVAT cohort was randomly divided into two samples for training (two-thirds, n = 2291) and internal validation (one-third, n = 1148). We checked the comparability of the two samples. The outcome was the time between the first anniversary of the transplantation and the patient’s death with a functioning graft (right-censoring of returns to dialysis). We used a semi-parametric Cox model to construct the score [18]. Univariate analyses were performed in order to make a first variables selection (p<0.20, training sample). If the log-linearity assumption was unconfirmed, the variable was categorized according to thresholds traditionally used in the literature. The selected variables were further analyzed in a multivariable model. The less significant parameters were progressively removed (p<0.05, training sample). Thereafter, clinical relevant interactions were tested. The proportional hazard (PH) assumption was evaluated using weighted residuals analysis [19]. In order to obtain predicted probabilities of death for a patient, we finally estimated the corresponding parametric PH model based on log-likelihood maximization and Weibull distribution (a generalized Weibull distribution was not significantly better, likelihood ratio test, p = 0.92). The score corresponded to the normalized linear predictor of this final model. Calibration curves were generated by plotting non-parametric observed survival probabilities obtained with the Kaplan-Meier estimator against the mean individual predicted survival probabilities stratified on predicted risk of death. In order to estimate the net survival curves [20], we used the estimator proposed by Perme, Stare and Estève [21]. Expected mortality was computed from the lifetime tables available in the human mortality database (www.mortality.org). Statistical analyses were performed using software R version 3.0.1 with *ROCt*, *nricens*, *pec* and *relsurv* packages [22].

#### The evaluation of the prognostic capacities of the scoring systems

Time-dependent Receiver Operating Characteristic (ROC) curves were estimated [23] from the DIVAT validation sample and the STCS cohort. The 95% confidence interval of the area under the ROC curve (AUC) was non-parametrically obtained from 1000 bootstrap replications and by using the 2.5^th^ and the 97.5^th^ percentiles. The Harrell’s C-index for censored survival data was also estimated [24, 25] and reported in the S2 Table. Net Reclassification Improvement (NRI) was applied to evaluate the percentage of patient correctly reclassified into two risk categories [26]: we chose to define, in an arbitrary manner, the low-risk group by patients with a 5% cumulative probability of death at 10 years since the first anniversary of the transplantation. This threshold was defined since it seemed an acceptable value for reducing the follow-up for low-risk patients or, by contrast, increasing the follow-up frequency for high-risk patients.

## Results

### The French DIVAT cohort characteristics

Among the 2291 KTR of the training sample (Table 1 and Table 2), the mean age was 49.4 (±13.2) years and 62.1% were men. The mean time on dialysis before transplantation was 3.9 (±4.3) years. History of high blood pressure, cardiac angina and past history of diabetes concerned respectively 82.5%, 9.5% and 9.6% of the recipients before transplantation. The mean donor age was 48.4 (±15.5), 59.3% were men, 12.6% had a creatinine greater than 132 μmol/L and 51.2% cause of death was vascular brain damage. The median follow-up was 4.5 years (interquartile range from 2.4 to 7.1). At one year post transplantation, the mean recipient serum creatinine was 140.0 (±55.7) μmol.L^-1^. About 10% of patients had a NODAT in the first post transplantation year and 7.2% had a 1-year proteinuria higher than 1 g/24h. At 10 years since the first anniversary of the transplantation, 249 KTR of the training sample returned to dialysis, 129 died with a functioning graft and 185 remained alive. Regarding the 1148 KTR of the validation sample, 121 patients returned to dialysis, 69 died and 83 were still followed.

**Table 1:** Recipient, transplantation and donor quantitative characteristics at transplantation or at 1-year of transplantation of the training and validation samples (mean, standard deviation and missing values).

Characteristics	Training cohort (n = 2 291)			Validation cohort (n = 1 148)		
	Missing*	Mean	SD	Missing*	Mean	SD
**Recipient age** (years)	0 (0.0)	49.39	13.17	0 (0.0)	50.09	12.95
**Recipient BMI** (kg/m^2^)	17 (0.7)	23.83	4.29	7 (0.6)	23.98	4.24
**Waiting time on list** (years)	218 (9.5)	2.17	2.15	102 (8.9)	2.22	2.36
**Time on dialysis** (years)	0 (0.0)	3.89	4.25	0 (0.0)	3.87	4.20
**Cold ischemia time** (hours)	32 (1.4)	20.00	9.32	14 (1.2)	19.64	9.41
**Donor age** (years)	12 (0.5)	48.35	15.52	4 (0.3)	47.98	15.74
**Donor serum creatinine** (μmol.L^-1^)	81 (3.5)	93.59	53.59	41 (3.6)	93.28	52.10
**1-yr serum creatinine** (μmol.L^-1^)	0 (0.0)	139.60	55.69	0 (0.0)	137.80	54.83

Abbreviations: SD, Standard Deviation; BMI, Body Mass Index.

*Missing (effective and %).

**Table 2:** Recipient, transplantation and donor qualitative characteristics at transplantation or at 1-year of transplantation of the training and validation samples (effective, standard deviation and missing values).

Characteristics	Training cohort (n = 2 291)				Validation cohort (n = 1 148)			
	Missing*		Effective	%	Missing*		Effective	%
**Recipient male gender**	0	0.0	1 422	62.1	0	0.0	716	62.4
**Rank of the graft ≥ 2**	0	0.0	507	22.1	0	0.0	259	22.6
**Recurrent disease**	1	0.1	806	35.2	1	0.1	394	34.4
**Hemodialysis**	1	0.1	2 082	90.9	0	0.0	1 047	91.2
**Positive CMV serology**	9	0.4	1 381	60.5	3	0.3	705	61.6
**Positive HCV serology**	0	0.0	124	5.4	0	0.0	68	5.9
**Diabetes**	0	0.0	219	9.6	0	0.0	113	9.8
**High blood pressure**	0	0.0	1 889	82.5	0	0.0	918	80.0
**Cardiovascular**	0	0.0	880	38.4	0	0.0	453	39.5
**Angina**	0	0.0	217	9.5	0	0.0	101	8.8
**Dyslipidaemia**	0	0.0	633	27.6	0	0.0	335	29.2
**Neoplasia**	0	0.0	196	8.6	0	0.0	96	8.4
**Number of HLA-A/B/DR mismatches >4**	70	3.1	286	12.9	35	3.0	134	12.0
**NODAT**	0	0.0	224	9.8	0	0.0	128	11.1
**1-yr maintenance therapy with Tacrolimus**	0	0.0	1 529	66.7	0	0.0	757	65.9
**1-yr 24h-proteinuria > 1g**	0	0.0	164	7.2	0	0.0	78	6.8
**Donor male gender**	20	0.9	1 346	59.3	12	1.0	708	62.3
**Deceased donor**	5	0.2	2 094	91.6	2	0.2	1 032	90.1
**Vascular death of the donor**	34	1.5	1 155	51.2	25	2.2	555	49.4
**Donor positive CMV serology**	13	0.6	1 164	51.1	6	0.5	574	50.3
**Donor positive EBV serology**	278	12.1	1 862	92.5	127	11.1	942	92.6

Abbreviations: CMV, cytomegalovirus; HCV, Hepatitis C Virus; HLA, Human Leukocyte Antigen; NODAT, New Onset Diabetes After Transplantation; EBV, Epstein-Barr Virus.

*Missing (effective and %).

### The Swiss STCS cohort characteristics

Among the 800 KTR, the mean age was 51.3 (±13.7) years and 65.6% were men. The mean time on dialysis before transplantation was 3.5 (±5.2) years. History of cardiac angina and diabetes concerned respectively 11.5% and 13.3% of the recipients. The median follow-up was 2.9 years (interquartile range from 1.9 to 3.8). At one year post transplantation, the mean recipient serum creatinine was 133.0 (±49.4) μmol.L^-1^. At 4 years since the first anniversary of the transplantation, 21 recipients of the sample returned to dialysis, 31 died with a functioning graft and 2 were still followed. Except for the duration of the follow-up and the sample size, the Swiss KTR therefore presented a similar profile to the French ones. Patient characteristics of both the French and the Swiss cohorts are compared in the S3 Table.

### The construction of the 1-year scoring system

The aim of this subsection was to study the possibility of a new mortality risk score taking into account parameters during the first year of transplantation. The survival model obtained from the DIVAT training sub-cohort (n = 2291) is described in Table 3. As expected, recipient age at transplantation was significantly associated with an excess of mortality post transplantation. In accordance with the RRS of Baskin-Bey et al. [10], this association seemed different according to the diabetes status of recipients (p = 0.012). Prolonged waiting time on dialysis before transplantation, past history of cardiovascular events and high value of the 1-year serum creatinine were also associated with higher mortality (p<0.05). Interestingly, with the exception of the 1-year serum creatinine, all these risk factors were also retained in the RRS proposed by Baskin-Bey et al. [10]. Therefore, the model described in Table 3 can be qualified as an updated RRS for KTR with a functioning graft at 1-year post transplantation. This score is defined as the normalized linear predictor of the model, as follows:

1-yearRRS={[0.629*recipient age at transplantation(10years)+

3.853*(1ifpre-transplantdiabetesand0otherwise)+

$$0.220\;*\;\text{time on dialysis}\;(\sqrt{\mathrm{years}})+$$

$$0.113\;*\;\text{value of the}\;1-\text{year serum creatinemia}\;(\sqrt{\mu mol.L^{-1}})+$$

0.465*(1ifhistoryofcardiovasculareventand0otherwise)-

0.608*recipient age at transplantation(10years)*(1ifpre-transplantdiabetesand0otherwise)]−5.06}/0.99

**Table 3:** Results of the multivariable parametric survival model from the training sample (n = 2291). The scale and the shape parameters of the baseline Weibull hazard function were respectively equaled 2635.742 (SD = 0.671) and 1.318 (SD = 0.073).

	Log HR. (SD)	HR	95%CI	p-value
**Recipient age** (10 years) for non-diabetics patients at transplantation	0.629 (0.091)	1.88	[1.57–2.24]	<0.001
**Recipient age** (10 years) for diabetics patients at transplantation	0.021 (0.227)	1.02	[0.65–1.59]	0.927
**Time on dialysis** (√years)	0.220 (0.091)	1.25	[1.04–1.49]	0.016
**History of cardiovascular event** (Presence/Absence)	0.465 (0.183)	1.59	[1.11–2.28]	0.011
**Recipient 1-year serum creatinine** (√μmol.L^-1^)	0.113 (0.043)	1.12	[1.03–1.22]	0.008

Abbreviations: SD, Standard Deviation; HR, Hazard Ratio; CI, Confidence Interval.

Thus, a patient with a negative/positive value can be considered to have a post-transplant risk of death lower/higher than the mean risk observed. Furthermore, approximately 95% of patients have a score between -2 and 2, and 70% have a score between -1 and 1.

### The prognostic capacities of the scoring systems

**Fig 2:**
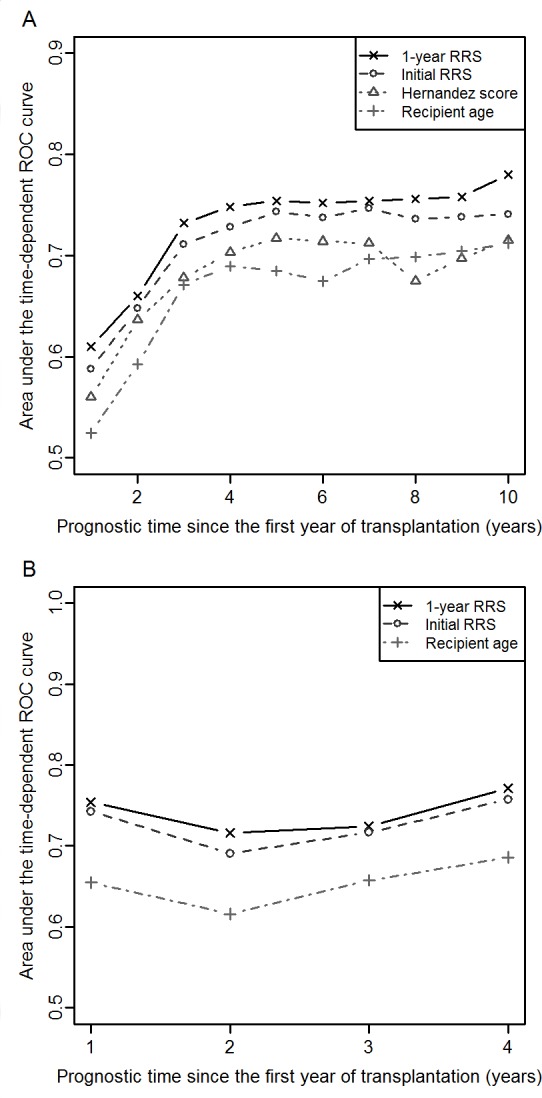
Area under time-dependent ROC curves according to prognostic time to evaluate the prognostic capacities of the different markers. (A) The analyses were based on the DIVAT validation sample (n = 1148). The 1-year updated RRS (black continuous line, 10-year AUC = 0.78, 95%CI = [0.69, 0.85]). The RRS (black dashed line, 10-year AUC = 0.74, 95%CI = [0.66, 0.80]). The score proposed by Hernandez et al. (grey dotted line, 10-year AUC = 0.71, 95%CI = [0.64, 0.78]). The recipient age (grey dashed-dotted line, 10-year AUC = 0.71, 95%CI = [0.64, 0.77]). (B) The analyses were based on the STCS cohort (n = 800). The 1-year updated RRS (black continuous line, 4-year AUC = 0.77, 95%CI = [0.68, 0.85]). The RRS (black dashed line, 4-year AUC = 0.76, 95%CI = [0.66, 0.83]). The recipient age (grey dashed-dotted line, 4-year AUC = 0.69, 95%CI = [0.61, 0.76]).

By using the DIVAT validation sample (n = 1148), Fig 2A shows the areas under ROC curves associated with the updated 1-year RRS, the RRS proposed by Baskin-Bey et al., the scoring system proposed by Hernandez et al., and the recipient age as single predictor. The prognostic capacities were rather constant for a prognostic time in between 3 and 10 years since the first anniversary of the transplantation. For the maximal prognostic time up to 10 years, the AUCs were respectively 0.78 (95%CI = [0.69, 0.85]), 0.74 (95%CI = [0.66, 0.80]), 0.71 (95%CI = [0.64, 0.78]), and 0.71 (95%CI = [0.64, 0.77]). The prognostic capacities of the 1-year RRS in terms of AUC differences were significantly higher than the recipient age (p = 0.03) and the score proposed by Hernandez et al. (p = 0.04), while they only tended to be higher than initial RRS (p = 0.10). This trend may be due to the low statistical power tied up to the small number of deaths (n = 69). Similar results were obtained by using the NRI. Note that we have additionally performed stratified analyses according to the donor status, which demonstrated the robustness of the RRS applied to living donor patients, even if it was initially developed for deceased donors.

By using the STCS cohort (Fig 2B, n = 800), 4 years was the maximum prognostic time with sufficient recipients still at-risk. The AUC related to the updated 1-year RRS for a prognostic up to 4 years was 0.77 (95%CI = [0.68, 0.85]). No significant difference was highlighted compared with the RRS (p = 0.31). The prognostic capacities of the Hernandez score could not be evaluated from the 800 Swiss KTR since some variables of the score were not available in the database.

### Thresholds definition for medical decision-making

**Fig 3:**
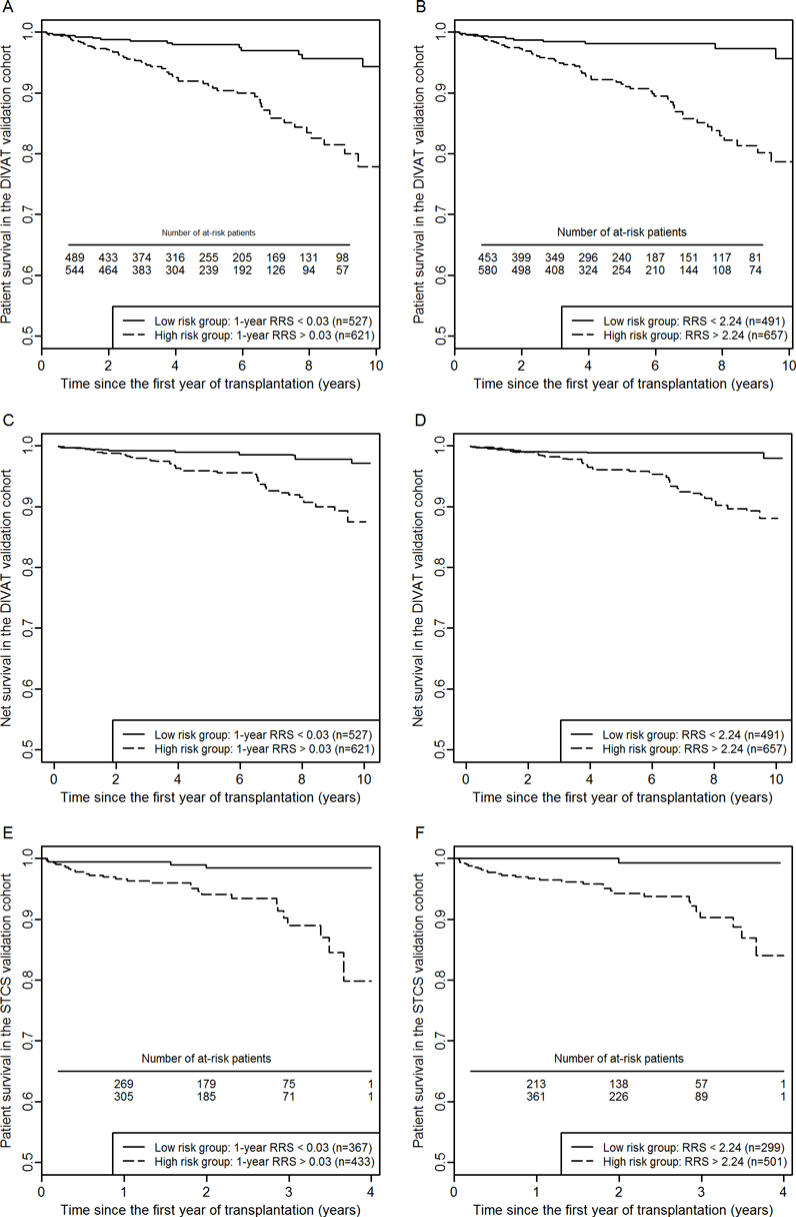
**Patient survival curves according to the two groups defined by the 1-year RRS threshold at 0.03 (A, C, E) or the RRS threshold at 2.24 (B, D, F).** (A, B) The analyses concerned the all-cause mortality in the DIVAT validation sample (n = 1148). (A) The black continuous line corresponds to the low-risk group with a 95.1% probability of being still alive with a functioning graft at 10 years since the first anniversary of the transplantation (95%CI = [90.7, 98.0]). The survival of the high-risk group was estimated at 77.9% (95%CI = [74.0, 85.4]). The difference between the two curves is highly significant (log-rank test, p<0.001). (B) The survivals of the low-risk group and the high-risk group were estimated at respectively 95.6% (95%CI = [91.9, 99.5]) and 78.7% (95%CI = [72.7, 85.1]) (log-rank test, p<0.001). (C, D) The analyses concerned the kidney transplant recipients’ related mortality in the DIVAT validation sample. (C) The black continuous line corresponds to the low-risk group with a 97.8% probability (95%CI = [95.1, 100.0]). The survival of the high-risk group is lower with a probability estimated at 89.3% (95%CI = [82.8, 96.3]). (D) The net survivals of the low-risk group and the high-risk group were estimated at respectively 98.0% (95%CI = [94.1, 100.0]) and 88.1% (95%CI = [81.3, 95.3]) (E, F) The analysis concerned the all-cause mortality in the STCS cohort (n = 800). (E) The black continuous line corresponds to the low-risk group with a 98.4% probability of being still alive with a functioning graft at 4 years since the first anniversary of the transplantation (95%CI = [96.8, 100.0]). The survival of the high-risk group was estimated at 81.3% (95%CI = [72.1, 91.7]). The difference between the two curves is highly significant (log-rank test, p<0.001). (F) The survivals of the low-risk group and the high-risk group were estimated at respectively 99.3% (95%CI = [97.9, 100.0]) and 84.0% (95%CI = [76.3, 92.6]) (log-rank test, p<0.001).

For the usefulness of the RRS and the 1-year RRS, it appeared important to suggest thresholds which could help physicians in medical decision-making, in particular for follow-up adaptation. (Fig 3) We estimated from the DIVAT training sample (n = 2291) that a cut-off at 0.03 for the 1-year RRS and 2.24 for the RRS corresponded to a negative predictive value at 0.95. In the DIVAT validation sample (n = 1148), we confirmed that patients with a 1-year RRS lower than 0.03 (n = 527, 46%) or a RRS lower than 2.24 (n = 491, 43%) had less than 5.0% risk of death up to 10 years since the first anniversary of the transplantation (Fig 3A and 3B). By removing the expected mortality of a similar population from the general French population with same gender, age and birth date, the risk of death related to the kidney transplantation status was 2.2% in the low-risk group defined by the 1-year RRS (Fig 3C) and 2.0% in the low-risk group defined by the RRS (Fig 3D). By using the external STCS validation sample (n = 800), patients with a 1-year RRS lower than 0.03 had 1.6% risk of death up to 4 years since the first anniversary of the transplantation (Fig 3E, 95%CI = [0.00, 0.03]) and 0.7% for patients with a RRS lower than 2.28 (Fig 3F: 95%CI = [0.00, 0.02]).

In the DIVAT validation sample, a 1-year RRS value higher than 0.03 was associated with an all-cause cumulative probability of death at 10 years since the first anniversary of the transplantation at 0.22 (Fig 3A, 95%CI = [0.15, 0.29]). This probability was 0.21 for patients with a RRS value higher than 2.24 (Fig 3B, 95%CI = [0.15, 0.27]). In the Swiss validation sample, these probabilities at 4 years were respectively 0.19 (Fig 3E, 95%CI = [0.08, 0.28]) and 0.16 (Fig 3F, 95%CI = [0.07, 0.24]).

### Calibration analyses of the 1-year RRS for individual predictions

**Fig 4:**
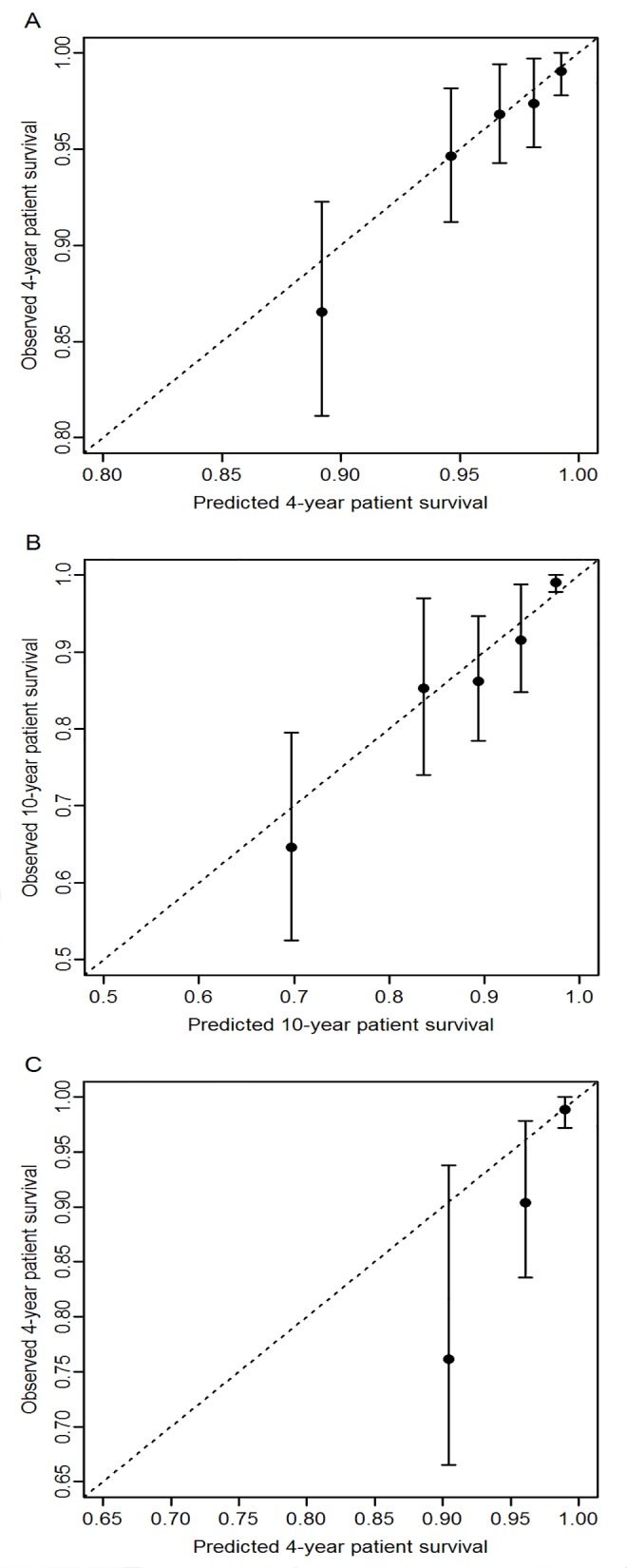
Calibration curves of the observed patient survival according to the predicted patient survival based on the 1-year RRS. (A) Patient survival was estimated from the DIVAT internal validation sample (n = 1148) at 4 years since the first anniversary of the transplantation. The sample was divided into 5 risk groups defined by the predicted patient survival. (B) Patient survival was estimated from the DIVAT internal validation sample (n = 1148) at 10 years since the first anniversary of the transplantation. The sample was divided into 5 risk groups. (C) Patient survival was estimated from the STCS cohort (n = 800) at 4 years since the first anniversary of the transplantation. The sample was divided into 3 risk groups since the number of deaths in the STCS cohort is low.

The mean predicted probabilities of death were very close to the observed ones at 4 and 10 years since the first anniversary of the transplantation by using the DIVAT validation sample (Fig 4A and 4B, respectively). For the calibration analyses from the Swiss cohort (Fig 4C), the predicted probabilities of death were also similar for the patients with the lowest risk of death. In contrast, the 1-year RRS model was associated with an underestimation of the risk of death for the other two thirds of the recipients. This underestimation was also observable by comparing the patient survival curves for the high-risk patients with a 1-year RRS higher than 0.3 in both French and Swiss validation samples (Fig 3A and 3E).

## Discussion

In kidney transplantation, both prognoses of return to dialysis and death could help to establish a more personalized follow-up. We believe that the first anniversary of the transplantation is a relevant landmark to decide the follow-up frequency. On the one hand, the evaluation of the risk to return to dialysis can be achieved by using the KTFS or other scoring systems [8, 27, 28]. The KTFS is based on 8 clinical and biological factors collected within the first year of transplantation and aims to stratify patients according to their risk of return to dialysis. It is currently used in a French clinical trial in which the follow-up frequency is proposed according to the KTFS estimation [7]. On the other hand, concerning mortality prediction, we firstly focused in this paper on developing a new scoring system computable at 1-year post transplantation. We secondly studied its prognostic capacities in comparison with the ones obtained by two other scoring systems [9, 10].

This alternative scoring system we proposed is based on 5 parameters which are well-established as risk factors of post transplantation mortality [29–36] and close to the ones included in the initial RRS, except for the 1-year serum creatinine as an additional parameter. Notice that we did not use the estimated Glomerular Filtration Rate (eGFR) because the recipient age and gender were already tested as parameters of the score. Notice also that prognostic capacities of this updated 1-year RRS would have been certainly improve by taking into account patient reported outcomes, such as compliance, quality of life, stress or depression. However, such data were unavailable.

Based on the French DIVAT validation cohort, we demonstrated that the prognostic capacities of the 1-year RRS appeared to be significantly better than the ones of the scoring system proposed by Hernandez et al. [9]. Additionally, the results indicated that the 1-year RRS outperformed the recipient age used as single predictor. In accordance with Moore et al. [37], a score may have a true clinical utility if its prognostic capacities are higher than other simple and available metric(s), which is obviously the case in our application. Therefore, our results validated the prognostic capacities for French and Swiss recipients, i.e. for western European recipients.

Despite the previous validations of the prognostic capacities of the updated 1-year RRS, we were not able to demonstrate significantly better prognostic capacities than the initial RRS. It constitutes an additional result in favor of the initial of the pre-transplant RRS which can also be used at 1 year post-transplantation. Nevertheless, note that a limitation of our study is the large number of patients non-included (because of missing value for at least one variable of the prognostic scores) which is likely to decrease the statistical power.

It is also important to note that internal validation (French DIVAT cohort) was performed for a prognostic time at 10 years while external validation (Swiss STCS cohort) could be performed only at 4 years, owing to an insufficient follow-up duration of the Swiss patients. Therefore, the 1-year RRS may potentially be more appropriate for use in France and tentatively in Western Europe, but future studies on largest European KTR cohorts are required. Development and validations of the 1-year RRS were based on two Western European cohorts. Therefore its capacities for predicting post-transplantation deaths in countries with different patients’ characteristics may be challenged and other external validations are needed.

We proposed cut-offs in order to stratify patients into two groups. Patients with a RRS lower than 2.24 (or with a 1-year RRS lower than 0.03) can be considered at low-risk with a 10-year risk of dying being less than 5%. Based on these results, one can reasonably hypothesize that recipients classified at low-risk to return to dialysis (assessed for instance by a KTFS level lower than 4.17) and to die may benefit of a lightened follow-up after the first year post transplantation. These prediction capacities for low-risk patients were validated in terms of calibration in both internal and external validation samples. Nevertheless, it is important to notice that the risk of death was underestimated for Swiss patients classified in the high-risk group. Therefore, the overall results tended to demonstrate the usefulness of the RRS and the 1-year RRS to accurately identify patients with a low probability of death, the price to pay being a high percentage of false positive tests, acceptable if our objective is to reduce the follow-up for low-risk patients and maintain a traditional follow-up for others.

In addition to the adaptation of the follow-up frequency, we believe that the RRS can also be used to adapt the nature of consultations, i.e. by focusing on modifiable risk factors included in the RSS. High-mortality risk is particularly attributable to the past history of diabetes and cardiovascular diseases. The RRS calculation could encourage to enhanced efforts for cardio vascular risk reduction: better glycemia and blood pressure controls and/or reinforced pharmacologic therapy. It could also guide physicians to better prevent cardiovascular events with, for instance, specific educational or physical program activities.

In summary, we proposed an updated version of the RRS for patients with functioning graft at 1-year post transplantation. The latter was internally and externally validated, but no significant superiority was evidenced compared to the initial RRS. We also demonstrated the ability of the pre-transplant RRS for predicting mortality after the first anniversary of the kidney transplantation and its possible extension to living donor recipients. Therefore, the two scoring systems may constitute candidates to further increase the efficiency of kidney transplantation compared to long-term dialysis. Additional studies on larger cohorts should be important for further validations our results. The 1-year RRS can nevertheless be used from now by using the application available at www.divat.fr/en/online-calculators.

## Supporting Information

S1 File The entire DIVAT cohort composed of 3439 patients transplanted between 2000 and 2012 in 6 French centers. (XLSX)

S1 Table Comparative analysis of the variables used the scores according to the two following samples: patients without missing values for the variables used in the scores versus patients with a missing value for at least one variable used in the scores. (PDF)

S2 Table Prognostic capacities of the 4 scoring systems up to 10 years in the DIVAT validation sample and 4 years in the STCS cohort. (PDF)

S3 Table Comparative analysis of the baseline characteristics between the DIVAT and the STCS cohort. (PDF)
