# Supplementary material for: Mortality Prediction after the First Year of Kidney Transplantation: An Observational Study on Two European Cohorts
Source: PLoS One. 2016 May 6;11(5):e0155278. doi: 10.1371/journal.pone.0155278 (PMC4859488; doi:10.1371/journal.pone.0155278)
Supplement: S1 Table — (PDF) [file pone.0155278.s002.pdf]

S1 Table. **Comparative analysis of the variables used the scores according to the two following samples: patients without missing values for the variables used in the scores versus patients with a missing value for at least one variable used in the scores.**

	**Patients without missing values (n=3 439)**	**Patients with missing values for 1 variable of the scores (n=2 099)**		**p-value**
**Quantitative characteristics: mean ± SD**		Missing*		
Recipient age at transplantation (years)	49.62 ± 13.10	0	48.70 ± 13.65	0.192
Time on dialysis (years)	3.88 ± 4.24	564	4.05 ± 4.09	0.207
1-year serum creatinine (μmol.L^{-1})	138.34 ± 55.10	412	139.68 ± 60.75	0.428
**Categorical characteristics: effective (%).**				
Positive HCV serology	192 (5.6)	63	99 (4.9)	0.277
Pre-transplant diabetes	332 (9.7)	0	211 (10.1)	0.662
History of cardiovascular event	1333 (38.8)	0	828 (39.4)	0.369
History of cardiac angina	318 (9.2)	0	175 (8.3)	0.269
NODAT	352 (10.2)	0	215 (10.2)	0.990
1-year maintenance therapy with Tacrolimus	2286 (66.5)	0	1410 (67.2)	0.611
1-year 24h-proteinuria > 1g	242 (7.0)	1670	22 (5.1)	0.169

Abbreviations: NODAT, New Onset Diabetes After Transplantation
