# Supplementary material for: Mortality Prediction after the First Year of Kidney Transplantation: An Observational Study on Two European Cohorts
Source: PLoS One. 2016 May 6;11(5):e0155278. doi: 10.1371/journal.pone.0155278 (PMC4859488; doi:10.1371/journal.pone.0155278)
Supplement: S2 Table — (PDF) [file pone.0155278.s003.pdf]

S2 Table. Prognostic capacities of the 4 scoring systems up to 10 years in the DIVAT validation sample and 4 years in the STCS cohort.

	**DIVAT cohort validation sample (n=1148)**		**STCS cohort (n=800)**	
	10-year AUC [95%CI]	10-years Harrell's C-Index [95%CI]	4-years AUC [95%CI]	4-years Harrell's C-Index [95%CI]
Recipient age	0.71 [0.64, 0.77]	0.71 [0.64, 0.78]	0.69 [0.61, 0.76]	0.67 [0.59, 0.73]
Hernandez score	0.71 [0.64, 0.78]	0.70 [0.63, 0.77]	-	-
RRS	0.74 [0.66, 0.80]	0.75 [0.66, 0.82]	0.76 [0.66, 0.83]	0.75 [0.64, 0.83]
1-year RRS	0.78 [0.69, 0.85]	0.77 [0.68, 0.86]	0.77 [0.68, 0.85]	0.76 [0.68, 0.83]

AUC: Area under the time dependent ROC curve; CI: Confidence interval.
