# Supplementary material for: Mortality Prediction after the First Year of Kidney Transplantation: An Observational Study on Two European Cohorts
Source: PLoS One. 2016 May 6;11(5):e0155278. doi: 10.1371/journal.pone.0155278 (PMC4859488; doi:10.1371/journal.pone.0155278)
Supplement: S3 Table — (PDF) [file pone.0155278.s004.pdf]

S3 Table. **Comparative analysis of the baseline characteristics between the DIVAT and the STCS cohort.**

	**DIVAT cohort (n=3 439)**	**STCS cohort (n=800)**	**p-value**
**Quantitative characteristics: mean ± SD**			
Recipient age at transplantation (years)	49.62 ± 13.10	51.32 ± 13.71	0.066
Time on dialysis (years)	3.88 ± 4.24	3.54 ± 5.21	0.082
1-year serum creatinine ($\mu mol.L^{-1}$)	139.60 ± 55.66	133.05 ± 49.37	0.054
**Categorical characteristics: effective (%)**			
Recipient male gender	2138 (62.2)	525 (65.6)	0.075
Pre-transplant diabetes	332 (9.7)	106 (13.3)	0.003
History of cardiovascular event	1333 (38.8)	315 (39.4)	0.779
History of cardiac angina	318 (9.2)	92 (11.5)	0.061
